# Integrated Characterization of lncRNA-Immune Interactions in Prostate Cancer

**DOI:** 10.3389/fcell.2021.641891

**Published:** 2021-02-16

**Authors:** Wei Hu, Yanru Wang, Zhixiao Fang, Wei He, Shengli Li

**Affiliations:** ^1^Institute of Translational Medicine, Shanghai General Hospital, Shanghai Jiao Tong University School of Medicine, Shanghai, China; ^2^Department of Nuclear Medicine, Huadong Hospital Affiliated to Fudan University, Shanghai, China

**Keywords:** long non-coding RNA, immune checkpoint, immunotherapy, prostate cancer, tumor immunology

## Abstract

Prostate cancer is among the top mortality factors in male around the world. Long non-coding RNAs (lncRNAs) have been shown to play crucial roles in tumor biology and immunology. However, lncRNA-immune interactions have not yet examined in prostate cancer. Here, we performed integrated analysis to characterize lncRNA-immune interactions in prostate cancer through multidimensional aspects, including immune-related hallmarks, tumor immunogenomic signatures, immune-related biological processes, immune cells, and immune checkpoints. We dissected the dysregulation of lncRNAs and their clinical relevance in prostate cancer, such as RP11-627G23.1 and RP11-465N4.5. Immune-related hallmarks took up the major parts among top significant lncRNA-hallmark interactions. Our analysis revealed that TGF-β signaling pathway was the most frequent to associate with lncRNAs, which is a signature of immune response in cancer. In addition, immune response and its regulation were the most closely connected immunological processes with lncRNA, implying the regulatory roles of lncRNAs on immune response in prostate cancer. We found that memory resting CD4^+^ T cells were the most lncRNA-correlated immune cell. LINC00861 was found to be potentially intervening targets of immunotherapy for prostate cancer patients, which was significantly associated with PD-1 and CTLA4. Collectively, we offered a handy resource to investigate regulatory roles of lncRNAs on tumor immunology and the development of clinical utility of lncRNAs in prostate cancer.

## Introduction

Prostate cancer is the most common malignancy in male, especially in Western World (Ferlay et al., 2015; Ku et al., 2019; Siegel et al., 2020). Despite substantial advances in diagnosis and therapeutics in prostate cancer, it still ranks the first cause of cancer mortality of male in the United States, which caused 33,330 deaths in 2020 statistics (Siegel et al., 2020). Studies regarding molecular alterations of prostate cancer offered mounts of potential diagnostic and therapeutic targets, with non-coding RNAs playing important roles (Ku et al., 2019).

Transcriptome diversity and their connections with critical biological processes have been investigated in multiple cancer types, among which non-coding RNAs took a large part (Kahles et al., 2018; Hua et al., 2019; Li et al., 2019). Long non-coding RNAs (lncRNAs) are >200 nt RNA molecules with limited protein-coding capability, which have been once considered as transcriptional noises (Du et al., 2013; Cech and Steitz, 2014; Iyer et al., 2015). Studies have shown that lncRNAs exert their biological functions through various ways, including miRNA sponges (Hansen et al., 2013; Yuan et al., 2014; Marchese et al., 2017), decoys to bind proteins (Carpenter et al., 2013; Qian et al., 2016), scaffolds or guides to regulate protein-protein or protein-DNA interactions (Engreitz et al., 2013; Lee et al., 2016). LncRNAs have been shown to play important roles in human cancers (Iyer et al., 2015; Niknafs et al., 2016; Li S. et al., 2018; Li Z. et al., 2018), including prostate cancer (Hua et al., 2018, 2019). In particular, down-regulation of UCA1 (urothelial carcinoma associated 1) inhibited proliferation of prostate cancer cells by modulating MYO6 through sponging miR-143 (Yu et al., 2020). Tumor immune microenvironment (TIME) is among the key factors impacting the treatment response, especially immunotherapy (Fridman et al., 2017; Mouw et al., 2017; Thorsson et al., 2018). The infiltrated abundance of cytotoxic and helper T lymphocytes within tumor microenvironment has shown prognostic and clinical implications in multiple cancer types (Fridman et al., 2017). A variety of immunogenomic features have been shown to contribute to influencing TIME, including tumor mutation burden and DNA damage repair defects (Bryant et al., 2017; Vitkin et al., 2019). Among genitourinary malignancies, prostate cancer shows unique TIME profiles with different features of infiltrated immune cell populations and immunogenomic features (Dallos and Drake, 2018; Vitkin et al., 2019). Through integrated analysis of lncRNA and immune features across 33 different cancer types, Li Y. et al. (2020) demonstrated that lncRNAs were closely interacted with immune-related pathways and infiltrated immune cells in cancer. However, the landscape of aberrant lncRNAs and their interactions with immune features in prostate cancer have not been characterized.

In the present study, we dissected the dysregulation of lncRNAs in prostate cancer and their clinical relevance. To comprehensively characterize lncRNA-immune interactions in prostate cancer, we assessed the associations between lncRNA expression and various immune features, including immune-related hallmarks, tumor immunogenomic signatures, immune-related biological processes, tumor infiltrated immune cells, and immune checkpoints. Our analysis revealed close connections between prostate cancer (PRAD) differential lncRNAs and these immune features in prostate cancer and suggested the potential clinical utility of lncRNAs in immunotherapy for patients with prostate cancer.

## Materials and Methods

### Differential Expression Analysis

The read count profiles of genes in 18 The Cancer Genome Atlas (TCGA) cohorts with more than five paired adjacent normal samples were retrieved from the Genomic Data Commons (GDC) data portal^1^ (Grossman et al., 2016). Raw read counts were normalized to FPKM units (Fragments Per Kilobase of transcript per Million mapped reads). Raw read count matrices were then subject to DESeq2 (Love et al., 2014) for differential expression analysis of long non-coding genes. Genes with fold change >1.5 and false discovery rate (FDR) (Benjamini–Hochberg corrected *P*-value) <0.05 were considered to be significantly expressed in tumor samples.

### Risk Evaluation and Survival Analysis

Expression levels of individual differential lncRNAs across all prostate cancer samples were used to investigate the relations between expression variations and patient prognosis. The Cox univariate proportional hazards regression model was adopted to determine risk lncRNAs in prostate cancer. For each lncRNA, all patients were dichotomized into high- and low-expression groups using the median expression level as cut-off. The Kaplan–Meier algorithm was further utilized to compare overall survival times between the two groups as described in previous study (Li et al., 2017, 2019). Differences of overall survival times were estimated by using the log rank test.

### Calculation of Gene Set Scores

The hallmark gene sets were first obtained from the Molecular Signature Database (MSigDB) (Liberzon et al., 2015). Then, the hallmark scores in each sample were calculated based on Gene Set Variation Analysis (GSVA) (Hänzelmann et al., 2013). Specifically, the GSVA algorithm was employed to evaluate the variation of hallmark activities over each sample in an unsupervised manner by utilizing expression profiles of genes annotated in corresponding hallmarks. Finally, the activity score of each hallmark was assigned to each sample.

### Enrichment of lncRNAs in Biological Features

To evaluate the enrichment of individual lncRNAs in specific biological features, the correlations between individual lncRNAs and genes of biological features were first calculated by Spearman’s correlation. LncRNA-gene pairs with | Rs| > 0.3 and FDR < 0.05 were considered as significant correlated pairs. For each lncRNA, Fisher’s exact test and hypergeometric test was employed to estimate the difference of significant pairs between interested features and the others across 50 hallmarks and 95 immune-related biological processes, respectively. LncRNA-hallmark pairs with OR > 1 and FDR < 0.05 were considered as significantly enriched pairs. The corresponding lncRNAs were assigned to significant paired biological features as highly associated lncRNAs.

### Estimation of Immune Cell Abundance in Tumor Samples

For each sample, the CIBERSORT (Newman et al., 2015) algorithm was employed to evaluate the relative immune cell abundance from gene expression profiles. In particular, immune-cell-type gene expression was deconvolved based on predefined immune cell signatures. In this study, the LM22 immune cell signature was adopted. These immune cells were validated to differentially express in one certain leukocyte population compared to all other hematopoietic cell types. Specifically, LM22 signature includes 22 different immune cell types, i.e., “B cells naive,” “B cells memory,” “Plasma cells,” “T cells CD8,” “T cells CD4 naive,” “T cells CD4 memory resting,” “T cells CD4 memory activated,” “T cell follicular helper,” “T cells regulatory (Tregs),” “NK cells resting,” “NK cells activated,” “Monocytes,” “Macrophages M0,” “Macrophages M1,” “Macrophages M2,” “Dendritic cells resting,” “Dendritic cells activated,” “Mast cells resting,” “Mast cells activated,” “Eosinophils,” and “Neutrophils.” The correlation between expression level of each lncRNA and abundance of immune cells was then calculated to determine immune cell-related lncRNAs in prostate cancer.

### Statistical Analysis and Plots

All statistical calculation and plots in this study were performed in R environment^2^. Unless specially stated, a statistical test with *P*-value or FDR < 0.05 was considered as significant.

## Results

### Characterization of lncRNA Dysregulation and Clinical Relevance in Prostate Cancer

**FIGURE 1:**
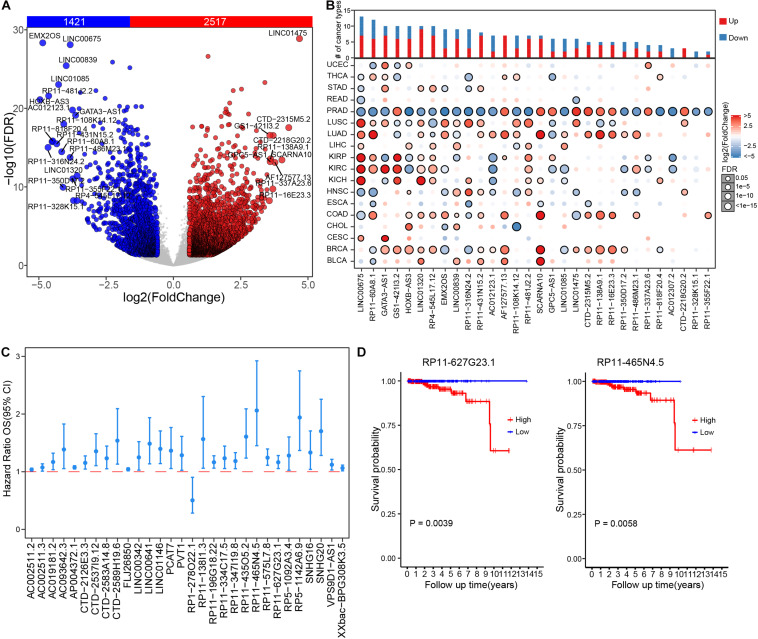
Dysregulation and clinical relevance of lncRNAs in prostate cancer. **(A)** Volcano plot shows the expression difference of lncRNA in prostate cancer samples compared to those paired adjacent normal samples. **(B)** The expression changes of top 30 differential lncRNAs of prostate cancer across 18 TCGA cancer types. **(C)** The survival risk assessment of differential lncRNAs, showing top significant lncRNAs (FPKM >1) in univariate regression analysis. **(D)** Kaplan–Meier curve of RP11-627G23.1 and RP11-465N4.5 in PRAD cohort.

To systematically investigate dysregulation of lncRNAs in prostate cancer, we retrieved gene expression matrix from TCGA PRAD cohort, including 499 tumor and 52 paired adjacent normal prostate samples. All lncRNAs annotated in GENCODE (release v22) were extracted from the obtained expression matrix of PRAD. In total, 13,676 lncRNAs were detected with expression of no less than 0.1 FPKM in at least one PRAD sample. Various expression cut-offs were used to examine the expression distributions of lncRNAs across PRAD samples. Despite universal low expression, lncRNAs were extensively expressed in PRAD samples. In particular, an average of 32.1% lncRNAs were detected in more than 90% samples and 22.7% lncRNAs were expressed in less than 10% samples (Supplementary Figure 1A). On average, 29.5% lncRNAs exhibited expression levels more than 0.1 FPKM, and 7.78% lncRNAs expressed more than 1 FPKM (Supplementary Figure 1B). Differential expression analysis was further performed, wherein 1,421 down-regulated and 2,517 up-regulated lncRNAs were identified in prostate cancer samples (Figure 1A and Supplementary Table 1). Most of PRAD differential lncRNAs were identified differential expression in multiple cancer types with over three quarters show differential expression in 4–10 cancer types (Supplementary Figure 1C and Supplementary Table 2). Across 18 different cancer types, 77 lncRNAs were exclusively differentially expressed in PRAD cohort. For example, RP11-328K15.1 showed no significant differential expression in multiple cancer types except PRAD (Figure 1B), which might indicate specific biological functions in the tumor biology of prostate cancer. To further investigate the clinical relevance of PRAD differential lncRNAs, associations between lncRNA expression and patient survival time were assessed by Cox regression analysis. The expression level of most differential lncRNAs were negatively associated with prognosis of PRAD patients, wherein seven lncRNAs were associated with good prognosis, such as RP1-278O22.1, while 128 lncRNAs were associated with bad prognosis, such as RP5-1142A6.9 (Figure 1C and Supplementary Table 3). Higher expression level of RP11-627G23.1 (*P* = 0.0039, log rank test) and RP11-465N4.5 (*P* = 0.0058, log rank test) were significantly associated with decreased survival of patients with PRAD (Figure 1D). Similarly, the expression level of most differential lncRNAs were also negatively associated with disease-free survival of PRAD patients (Supplementary Figure 2). Our results demonstrated that lncRNAs play important roles in PRAD and could be potential prognosis biomarkers.

### PRAD Differential lncRNAs Were Closely Associated With Biological Hallmarks

**FIGURE 2:**
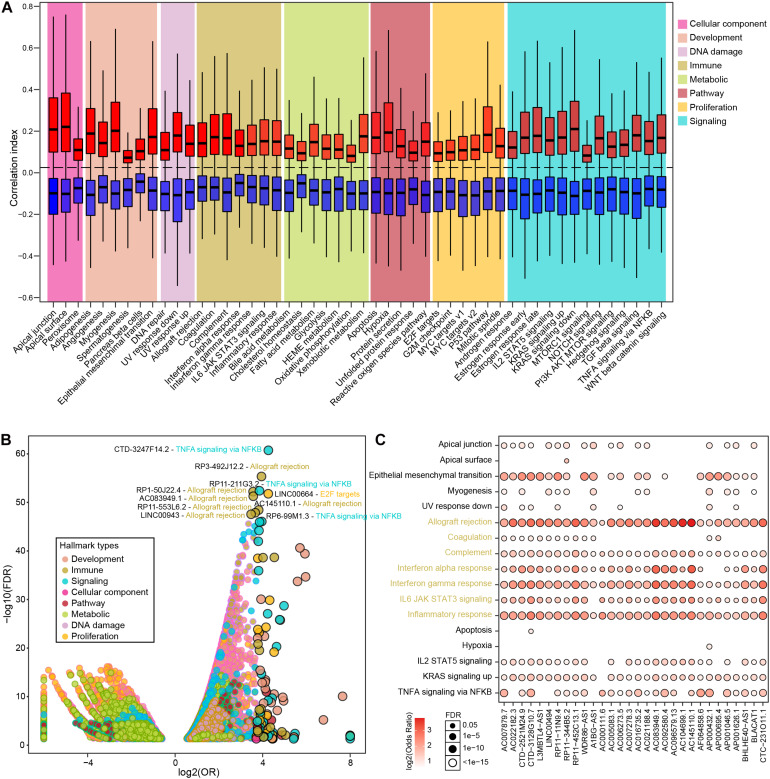
The correlations between PRAD differential lncRNAs and various hallmarks. **(A)** Boxplots show correlation indexes of positively and negatively correlated lncRNAs in each type of hallmark. **(B)** Bubble plot shows the enrichment of lncRNA-hallmark pairs in cancer hallmarks. **(C)** A subset of lncRNAs that were significantly enriched in immune-related hallmarks.

To further explore the major biological functions that differential lncRNAs might impact, we estimated the associations between 50 biological hallmarks and individual lncRNAs. Overall, differential lncRNAs tend to be more positively associated with biological hallmarks (Figure 2A), wherein the distribution of correlation index is relatively balanced associated in the immune hallmarks and less than one fifth exhibited significant correlations (Supplementary Figure 3A and Supplementary Table 4). We next performed enrichment analysis to examine lncRNAs that were exclusively correlated with some hallmarks than others (see section “Materials and Methods”). In total, 24,096 significant lncRNA-hallmark pairs were identified. Immune-related hallmarks showed relatively balanced distribution of highly associated lncRNA numbers among individual hallmarks, whereas other types exhibited larger number of associated lncRNAs in specific biological hallmarks (Supplementary Figure 3B and Supplementary Table 5). The most significantly enriched lncRNA-hallmark pair is between CTD-3247F14.2 and “TNFA signaling via NFKB.” The observation showed that CTD-3247F14.2 was prone to correlate with genes involved in “TNFA signaling via NFKB” than those in other hallmarks, indicated potential regulatory roles of CTD-3247F14.2 for “TNFA signaling via NFKB” in prostate cancer. Among the top 10 lncRNA-hallmark pairs, six are immune-related biological hallmarks, such as “allograft rejection” (Figure 2B). In addition, LINC00664 was exclusively significantly enriched in “E2F targets,” which is a proliferation-related hallmark. Among significant lncRNA-hallmark pairs, a subset of lncRNAs showed exclusively enrichment in most immune hallmarks. The majority of these lncRNAs were also enriched in the process of epithelial-mesenchymal transition, indicating potential lncRNA-mediated metastasis via immune processes (Figure 2C). Although cancer-related signaling and proliferative hallmarks showed the most associated lncRNAs, immune-related hallmarks hold almost half of the top significant enriched lncRNA-hallmark pairs (Supplementary Table 5).

### LncRNAs Showed Extensive Association With Immunogenomic Signatures in PRAD

**FIGURE 3:**
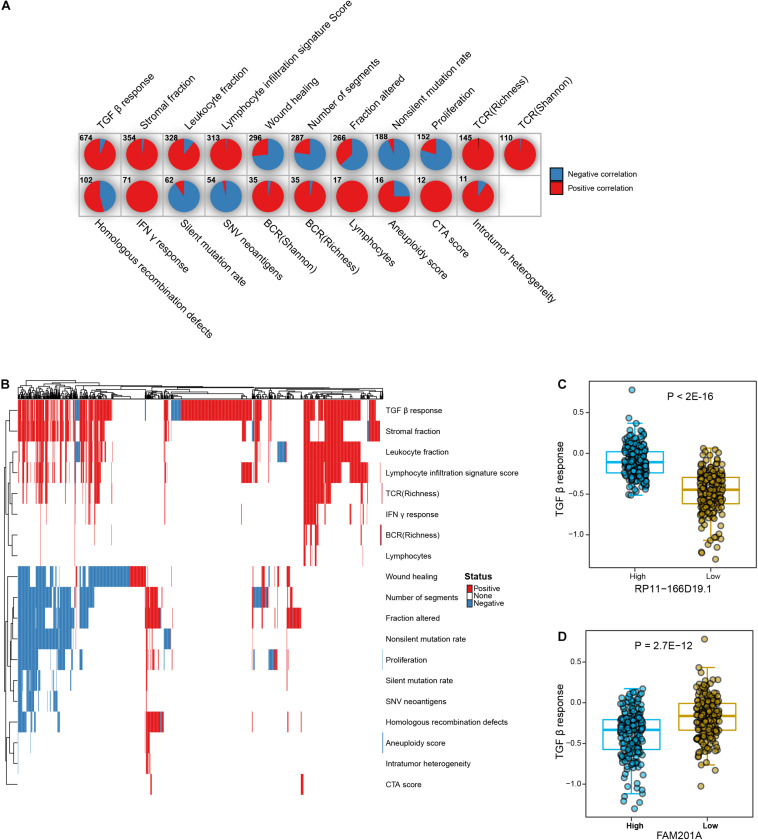
Associations between lncRNAs and tumor immune signature. **(A)** Pie charts show the numbers of significant positively and negatively correlated lncRNAs in each type of immunogenomic signatures. **(B)** Heatmap shows the landscape of significant correlated lncRNAs across different immunogenomic signatures. **(C)** Comparison of TGF-β response scores between high and low expression level of RP11-166D19.1 in PRAD samples. **(D)** Comparison of TGF-β response scores between high and low expression level of FAM201A in PRAD samples.

It is observed that a considerable portion of significantly enriched lncRNA-hallmark pairs were related to immune hallmarks, we next examined the associations between lncRNAs and immunogenomic signatures. Relative activities of 26 tumor immunogenomic signatures were retrieved from a previous study (Thorsson et al., 2018), which were utilized to assess the associations between lncRNAs and immunogenomic signatures in prostate cancer (see section “Materials and Methods”). The majority (21 in 26) of immunogenomic signatures have significantly associated lncRNAs, wherein most lncRNAs were positively associated with corresponding immunogenomic signatures (Figure 3A and Supplementary Table 6). “TGF-β response,” “stromal fraction,” “leukocyte fraction,” and “lymphocyte infiltration signature score” have larger number of associated lncRNAs (more than 300 lncRNAs). Besides some differential lncRNAs that were shared among distinct immunogenomic signatures, considerable proportions of lncRNAs were exclusively associated with individual immunogenomic signatures. In particular, almost half of “TGF-β response”-associated lncRNAs were specifically positive associated with “TGF-β response” (Figure 3B). Additionally, a subset of differential lncRNAs were exclusively negative associated with “wound healing” activity. Specifically, “TGF-β response” showed much higher activity in RP11-166D19.1-high PRAD samples than those in RP11-166D19.1-low PRAD samples (*P* < 2E-16, Figure 3C). Prostate cancer samples with higher level of FAM201A exhibited significantly lower level of “TGF-β response” activity (*P* = 2.7E-12, Figure 3D). In collection, our analysis suggested lncRNAs as markers of activity levels of immunogenomic signatures in prostate cancer.

### LncRNAs Were Frequently Connected With Immune-Related Biological Processes in Prostate Cancer

**FIGURE 4:**
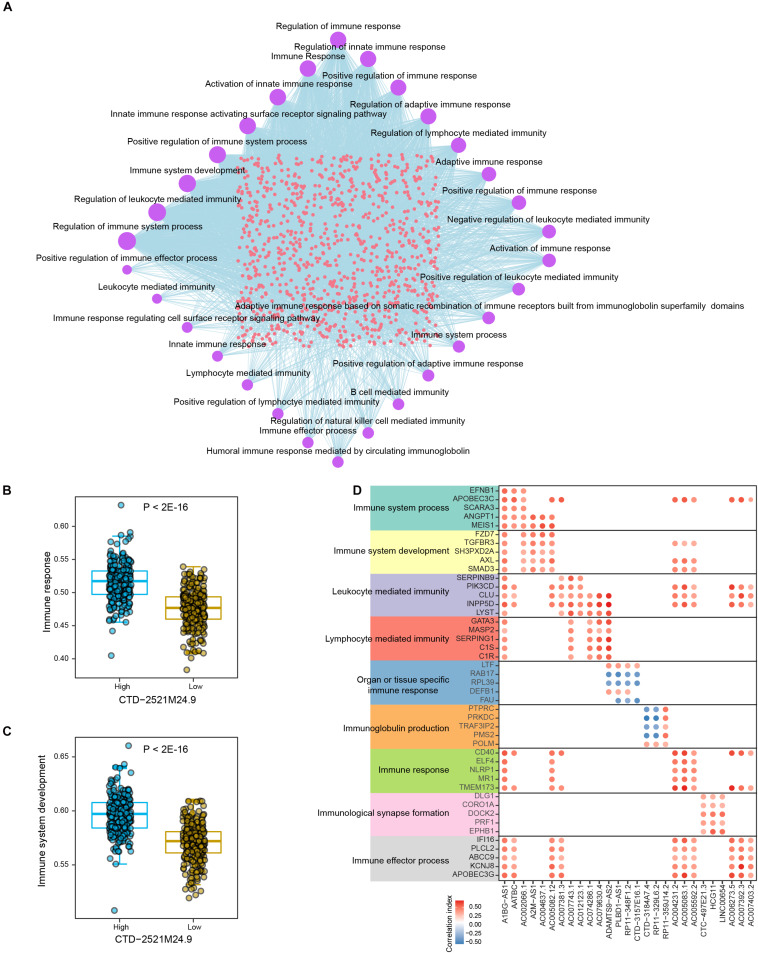
The crosstalk between lncRNAs and immune-related biological processes in PRAD. **(A)** Network shows the connections between differential lncRNAs and top significant immune-related biological processes in PRAD. Circle sizes indicate the numbers of significantly enriched lncRNAs for individual immune-related biological processes. **(B)** Comparison of immune response activities between high and low expression levels of CTD-2521M24.9. **(C)** Comparison of immune system development activities between high and low expression levels of CTD-2521M24.9. **(D)** Points show the correlations between lncRNAs and representative genes in individual immune-related biological processes.

We next explored the connections between lncRNAs and immune-related biological processes in prostate cancer. Totally, 38 immune-related processes were found to be significantly enriched by multiple lncRNAs in PRAD samples, such as “Regulation of immune response,” “Regulation of immune system process,” and “Immune system development” (Figure 4A and Supplementary Table 7). The most parts of differential lncRNAs potentially regulate immune response and immune system. In addition, differential lncRNAs were also found to be connected with cellular immunity, such as “Regulation of lymphocyte mediated immunity,” “leukocyte mediated immunity,” and “lymphocyte mediated immunity.” Prostate cancer samples with low level of CTD-2521M24.9 showed significantly lower activity level of immune response (*P* < 2E-16, Figure 4B). High expression level of CTD-2521M24.9 indicated high activity levels of immune system development (*P* < 2E-16, Figure 4C). Interestingly, different lncRNAs could regulate the same immune-related processes through modulating diverse genes (Figure 4D). For example, AC006273.5 was found to regulate “Immune response” by modulating CD40 and TMEM173, whereas A1BG-AS1 could potentially regulate “Immune response” through CD40, ELF4, NLRP1, MR1, and TMEM173. Furthermore, some lncRNAs exclusively regulate individual immune processes though the same genes. For example, LINC00654 were found to regulate “Immunological synapse formation” through DLG1, CORO1A, DOCK2, PRF1, and EPHB1. In summary, these observations demonstrated that lncRNAs were frequently connected with immune-related biological processes in prostate cancer.

### Interactions Between lncRNAs and Infiltrated Immune Cells and Immune Checkpoints Suggest Novel Therapeutic Strategy for Immunotherapy in Prostate Cancer

**FIGURE 5:**
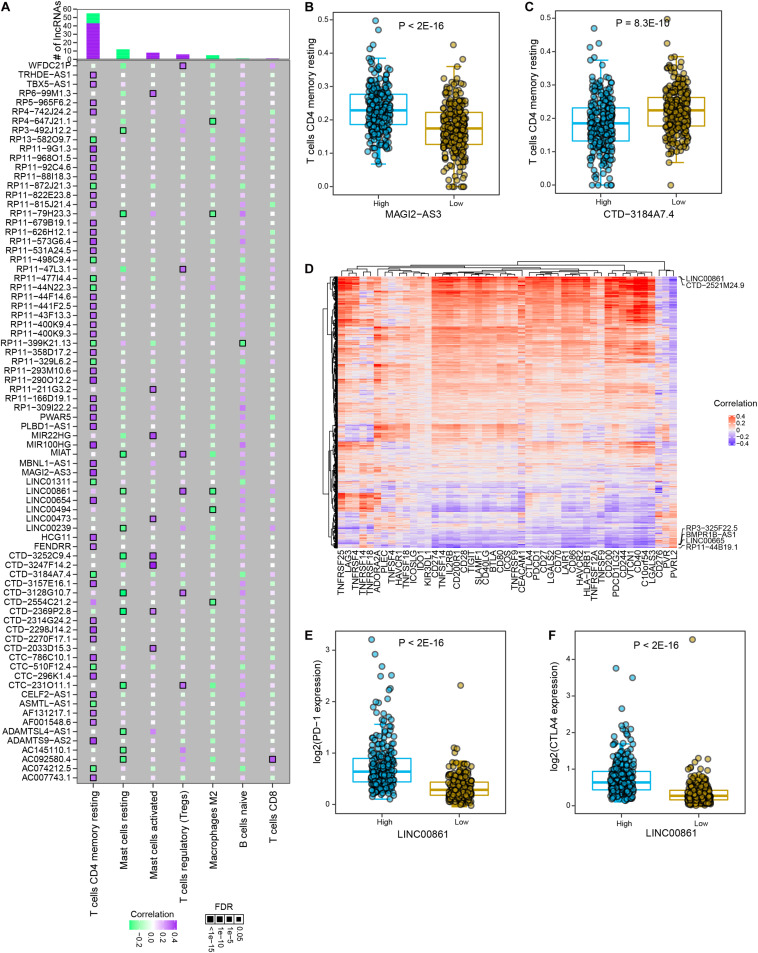
The interactions between PRAD differential lncRNAs and major immune cells and immune checkpoint genes. **(A)** The correlations between significant differential lncRNAs and major immune cell types. Colors indicate correlation coefficients and diamond sizes represent FDR values. **(B)** Comparison of relative abundance of memory resting T cells between high and low expression levels of MAGI2-AS3 in PRAD samples. **(C)** Comparison of relative abundance of memory resting T cells between high and low expression levels of CTD-3184A7.4 in PRAD samples. **(D)** Heatmap shows the correlations between the expression of immune checkpoint genes and differential lncRNAs. **(E)** Comparison of PD-1 expression between high and low LINC00861 expression levels in PRAD samples. **(F)** Comparison of CTLA4 expression between high and low LINC00861 expression levels in PRAD samples.

Studies on tumor immunology have proposed various therapeutic strategies for tumor patients, among which immune checkpoint blockade (ICB) therapy showed promising clinical benefits in multiple solid tumor types. Among significant lncRNA-immune cell interactions, memory resting CD4^+^ T cells were found to interact with the most differential lncRNAs, most of which were positively correlated (Figure 5A and Supplementary Table 8). In particular, cancer samples with high expression level of MAGI2-AS3 showed significantly higher infiltrated abundance of memory resting CD4^+^ T cells than those with low expression (*P* < 2E-16, Figure 5B). High expression of CTD-3184A7.4 indicated significantly lower level of infiltrated memory resting CD4^+^ T cell in prostate cancer (*P* = 8.3E-10, Figure 5C). To further explore the associations between lncRNAs and immune checkpoints in prostate cancer, we estimated the correlations between the expression levels of lncRNAs and immune checkpoint genes. Most of PRAD differential lncRNAs were found to be significantly positively correlated with immune checkpoint gene expression (Figure 5D and Supplementary Table 9). Some lncRNAs are positively associated with most immune checkpoint genes, such as LINC00861 and CTD-2521M24.9, while some are negative correlated with most of these genes, such as RP3-325F22.5, BMPR1B-AS1, LINC00665, and RP11-44B19.1. Specifically, LINC00861 was the most positively related to PD1 (*P* < 2E-16, Figure 5E), CTLA4 (*P* < 2E-16, Figure 5F) and TIGIT, and CTD-2521M24.9 was the most positively related to PD-L1; in contrast, LINC00665, RP3325F22.5, BMPR1B-AS1, and RP11-44B19.1 were the most negatively correlated to PD1, PD-L1, CTLA4, and TIGIT. Moreover, a Sankey diagram was employed to describe the connections between lncRNAs, immune cells and actionable immune checkpoints, with a subset of lncRNAs was closely related to the immune cells and actionable immune checkpoints (Supplementary Figure 4). These observations implied that LINC00861 might regulate the expression of PD-1 and CTLA4, actionable targets of ICB therapy or be efficient biomarker for their abundance in prostate cancer. Collectively, these results suggested that lncRNAs might be utilized to modulate activity of immune cells or levels of immune checkpoints to promote immunotherapy for patients with prostate cancer.

## Discussion

To comprehensively characterize the lncRNA-immune interactions in prostate cancer, our study explored the relations between lncRNAs and biological hallmarks, tumor immunogenomic signatures, immune-related biological processes, infiltrated immune cells, and immune checkpoints. Our study unveiled frequent interactions between PRAD differential lncRNAs and multiple immune features.

Our analysis presented comprehensive characterization of lncRNA dysregulation and clinical relevance in prostate cancer. We identified prostate cancer-specific dysregulated lncRNAs and prognostic lncRNAs, such as RP11-627G23.1 and RP11-465N4.5. We revealed the close associations between differential lncRNAs and biological hallmarks. Although immune-related hallmarks were not enriched of the largest number of lncRNAs, they took up approximately half of top significant lncRNA-hallmark associations. Allograft rejection is the top lncRNA-related cancer hallmarks, indicating the potential roles of dysregulated lncRNAs in organ transplantation. For example, in renal transplantation, oncogenic lncRNA-ATB is significantly overexpressed in acute rejection patients and regulates renal cell proliferation and cyclosporine A-mediated apoptosis (Qiu et al., 2017). The important roles of lncRNA-mediated innate and adaptive immune responses have been highlighted in recent years, especially in cancer immunity (Yu et al., 2018; Denaro et al., 2019; Wu et al., 2020). A subset of lncRNAs enriched in most immune hallmarks suggests their potential roles in modulating cancer immunity to facilitate cancer progression. For example, lncRNA cox-2 facilitates the polarization of M2 macrophages and therefore induces the malignant phenotypes of hepatocellular carcinoma cells and angiogenesis (Ye et al., 2018). As another example, FOXC1-mediated LINC00301 triggers malignant potential of non-small cell lung cancer cells and modulates the Tregs and CD8^+^ T cell populations by activating TGF-β signaling (Sun C.-C. et al., 2020).

Besides immune-related hallmarks, we also involved tumor immunogenomic signatures, immune-related biological processes, tumor infiltrated immune cells, and immune checkpoints. These features reflect immunological activities from diverse aspects in tumor samples (Grivennikov et al., 2010; Chen and Mellman, 2017; Greten and Grivennikov, 2019). In particular, tumor immunogenomic signatures represent genomic variations that were induced by or could induce immune reprogramming (Thorsson et al., 2018). Moreover, TGF-β signaling contributes to malignancy of cancer cells and immunosuppressive microenvironment, thus thwarting cancer immunotherapy (Colak and ten Dijke, 2017; Batlle and Massagué, 2019). A large amount of lncRNAs was related to TGF-β response in prostate cancer, suggesting their profound implication in TGF-β-mediated immune processes. For example, TGF-β1-simulated lncRNA DNM3OS induces transformation of prostate stromal cells by targeting miR-29a/29b/COL3A1 and miR-361/TGFβ1 axes (Wang et al., 2019). As another example, the therapeutic efficiency of PSMA-targeted human CAR T cells are enhanced upon TGF-β insensitivity in the treatment of prostate cancer (Kloss et al., 2018). Furthermore, stromal fraction is the second frequent lncRNA-related immune feature, profoundly implicating in cancer development and immunity (Tyekucheva et al., 2017; De Jaeghere et al., 2019; Ahn and Kim, 2020). For example, lncRNA H19 derived from carcinoma-associated fibroblasts (CAFs) contributes to the stemness and chemoresistance of colorectal cancer by targeting miR-141 (Ren et al., 2018). As another example, lncRNA-CAF induce transition from normal fibroblasts to CAFs by stabling IL-33, thereby leading to development of oral squamous cell carcinoma (Ding et al., 2018).

The activities of immune cells and abundance of immune checkpoints were crucial factors that affect the outcomes of immunotherapy for tumor patients (Cha et al., 2020; Jafari et al., 2020; Zhou et al., 2020). Our analysis found that a large number of lncRNAs were significantly associated with memory resting CD4^+^ T cells, such as MAGI2-AS3 and CTD-3184A7.4. Memory CD4^+^ T cell is a subset of T cell population that sustains in the absence of antigen and prepares for rapid immune response upon repeat antigen exposure (Hope et al., 2019). It has been shown that central memory CD4^+^ T cells in peripheral blood are associated with clinical response of PD-1 antibody therapy in melanoma patients. Our observations indicated that these lncRNAs might be able to modulate the activities of memory resting CD4^+^ T cells. Overexpression of MAGI2-AS3 or knockdown of CTD-3184A7.4 may activate memory resting CD4^+^ T cells to enhance immune response against tumor cells. Interestingly, MAGI2-AS3 has been widely studied in cancers, while these studies mainly focused in regulating cancer cells themselves (Liu et al., 2019; Li D. et al., 2020). Our results point out that various lncRNA could have immune regulatory role other than their influence on cancer cells. In addition, the close connection between lncRNAs and immune checkpoints has been revealed, suggesting a profound implication of lncRNA in immune checkpoint regulation. For example, LncRNA KCNQ1OT1 inhibits the cytotoxicity of CD8^+^ T cells and promotes the malignant ability of prostate cancer cells by targeting miR-15a/PD-L1 axis (Chen et al., 2020). As another example, lncAMPC activates LIF/LIFR/Jak1/STAT3 pathway to stable PD-L1 and metastasis-associated genes, thereby contributing to metastasis and immunosuppression in prostate cancer (Zhang et al., 2020). Of note, in prostate cancer, LINC00861 was closely associated with T cells regulatory, macrophages M2 and mast cells resting as well as a series of immune checkpoints, including PD1, PD-L1, and CTLA4. These evidence indicates a LINC00861-mediated tumor immune response beyond its reported regulation of malignant potential on cancer cells (Liu et al., 2021). Further experimental validation is needed to confirm the regulatory functions of the immune-related lncRNAs and select the most efficacious lncRNAs to boost the antitumor immune response. We believe that intervention of immune response through lncRNAs will be promising therapeutics for patients with prostate cancer.

## Conclusion

In conclusion, our study facilitated the understanding of lncRNA-immune interactions and provided a valuable resource of immune-related lncRNAs in prostate cancer. These lncRNAs could be potential biomarkers for immune cells or immune-related activities in prostate cancer. Because tumor immunity has major impacts on cancer progression, many immune-related lncRNAs can predict prognosis and immunotherapy of cancers (Zhou et al., 2017, 2018; Sun J. et al., 2020; Zhou et al., 2020). Therefore, these lncRNAs could be potentially utilized to predict and even modulate immune cell activities or immune checkpoint abundance to benefit immunotherapy for patients with prostate cancer.

## Data Availability Statement

The datasets presented in this study can be found in online repositories. The names of the repository/repositories and accession number(s) can be found in the article/Supplementary Material.

## Ethics Statement

The patient data we used were acquired as publicly available datasets that were collected with patients’ informed consent.

## Author Contributions

SL designed the study and wrote the manuscript. SL and WHe supervised the project. WHu, YW, and SL collected the data resource. WHu and SL performed the data analysis. WHu, YW, WHe, and ZF reviewed the manuscript. All authors read and approved the final manuscript.

## Conflict of Interest

The authors declare that the research was conducted in the absence of any commercial or financial relationships that could be construed as a potential conflict of interest.

## Footnotes

1 https://portal.gdc.cancer.gov/

2 https://www.r-project.org/

## Abbreviations

lncRNA: long non-coding RNA
TCGA: The Cancer Genome Atlas
PRAD: Prostate Adenocarcinoma
GDC: Genomic Data Commons
TIME: tumor immune microenvironment
ICB: immune checkpoint blockade
CAFs: carcinoma-associated fibroblasts
Tregs: regulatory T cells.
