# Supplementary material for: Integrated Characterization of lncRNA-Immune Interactions in Prostate Cancer
Source: Front Cell Dev Biol. 2021 Feb 16;9:641891. doi: 10.3389/fcell.2021.641891 (PMC7921328; doi:10.3389/fcell.2021.641891)
Supplement: Supplementary file 1 [file Table_1.docx]

**Supplemental Tables**

**Supplemental Table S1.** Differential lncRNAs in prostate cancer.

**Supplemental Table S2.** The expression difference of PRAD differential lncRNAs in multple cancer types.

**Supplemental Table S3.** Survival risk assessment of differential lncRNAs in PRAD.

**Supplemental Table S4.** Significantly correlated lncRNA-hallmark pairs.

**Supplemental Table S5.** The enrichment of lncRNAs in individual hallmark categories.

**Supplemental Table S6.** The correlations between lncRNAs and immunogenomic signatures.

**Supplemental Table S7.** The enrichment of lncRNAs in individual immune-related biological processes.

**Supplemental Table S8.** Associations between lncRNAs and abundance of infiltrated immune cells.

**Supplemental Table S9.** The correlations between lncRNAs and immune checkpoint genes.

**Supplemental Figures**


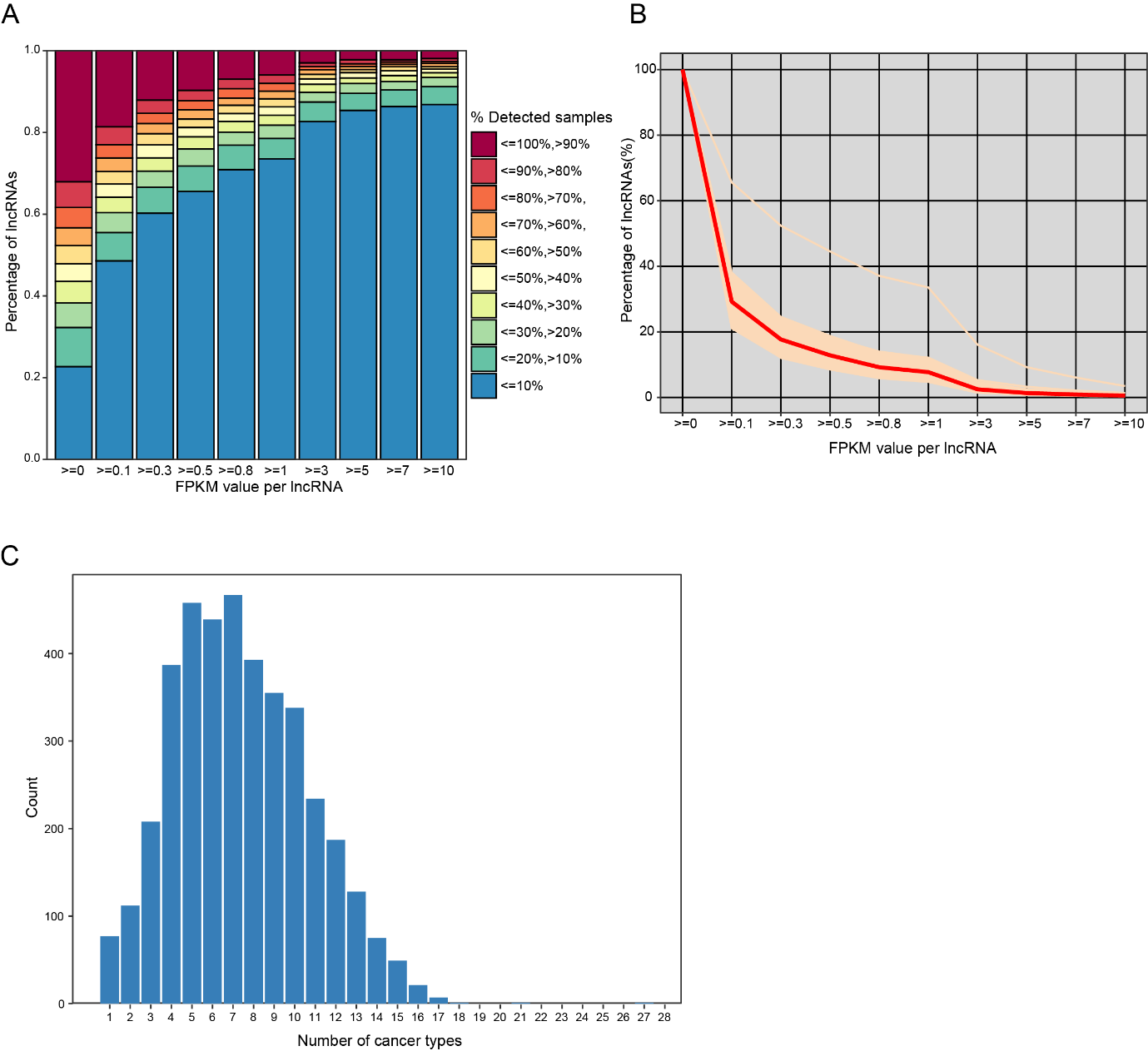


**Supplemental Figure 1.** **The transcriptional landscape of lncRNAs in prostate cancer samples. (A)** Detected sample distribution of lncRNAs with different expression range in prostate cancer samples. **(B)** Overall distribution of lncRNA expression levels in prostate cancer samples. **(C)** The distribution of cancer type numbers of expressed lncRNAs detected in prostate cancer samples.


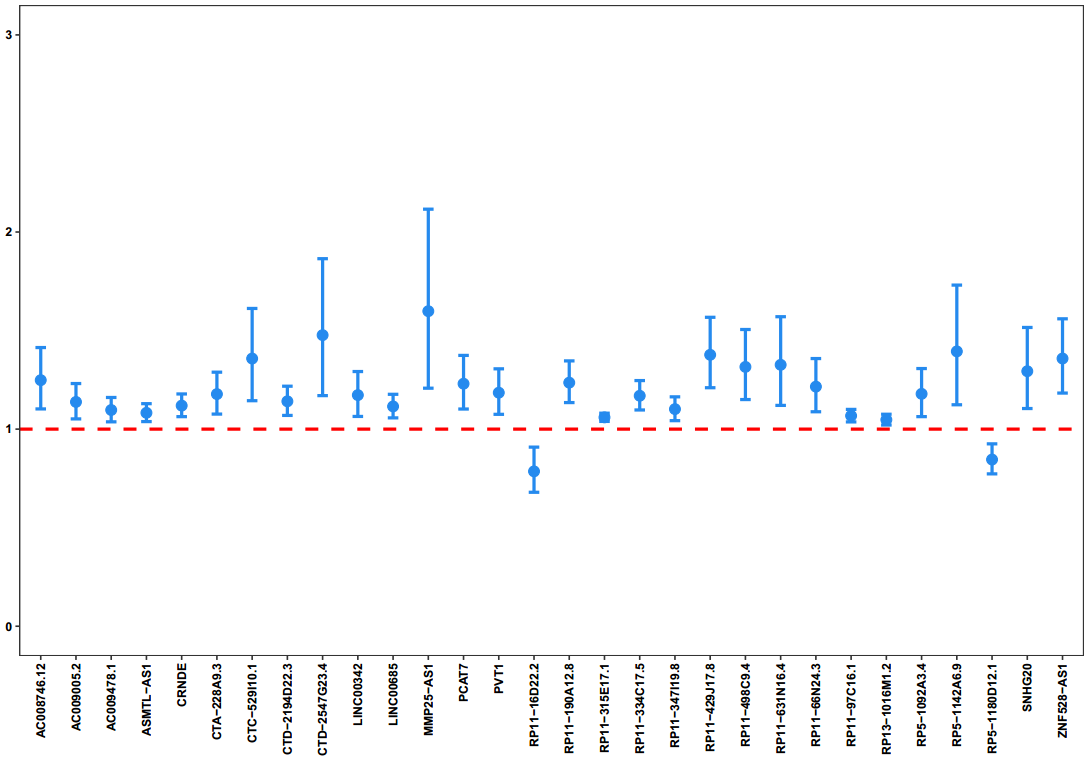


**Supplemental Figure 2.** The disease-free survival analysis of differential lncRNAs, showing FPKM>1 top significant lncRNAs in univariate regression analysis.


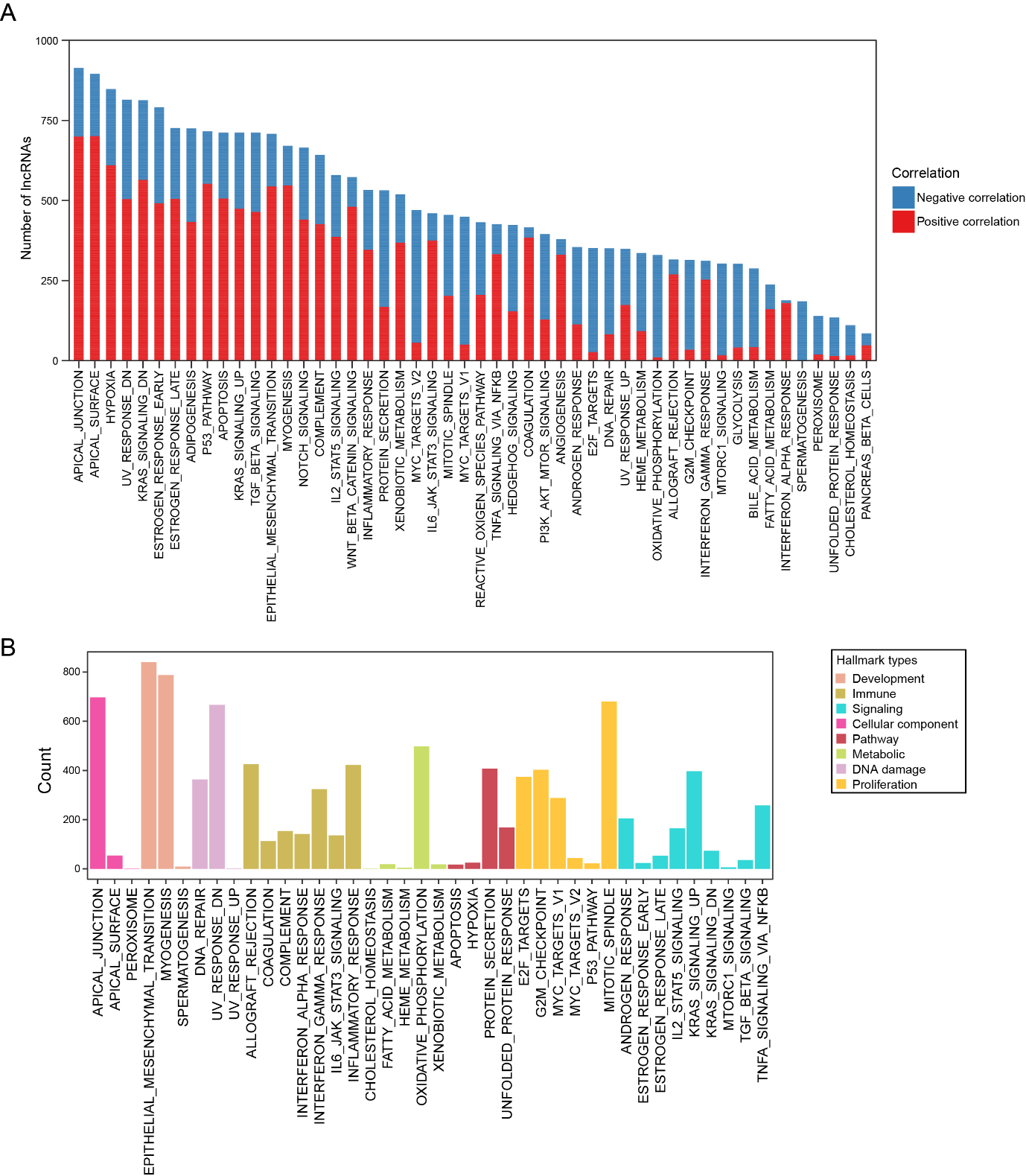


**Supplemental Figure 3.** The distribution of lncRNA-hallmark correlation and enrichment pairs**. (A)** The number distribution of hallmark-related lncRNAs across 50 hallmarks in prostate cancer patients. **(B)** The number distribution of lncRNAs that were enriched in individual hallmarks.

**
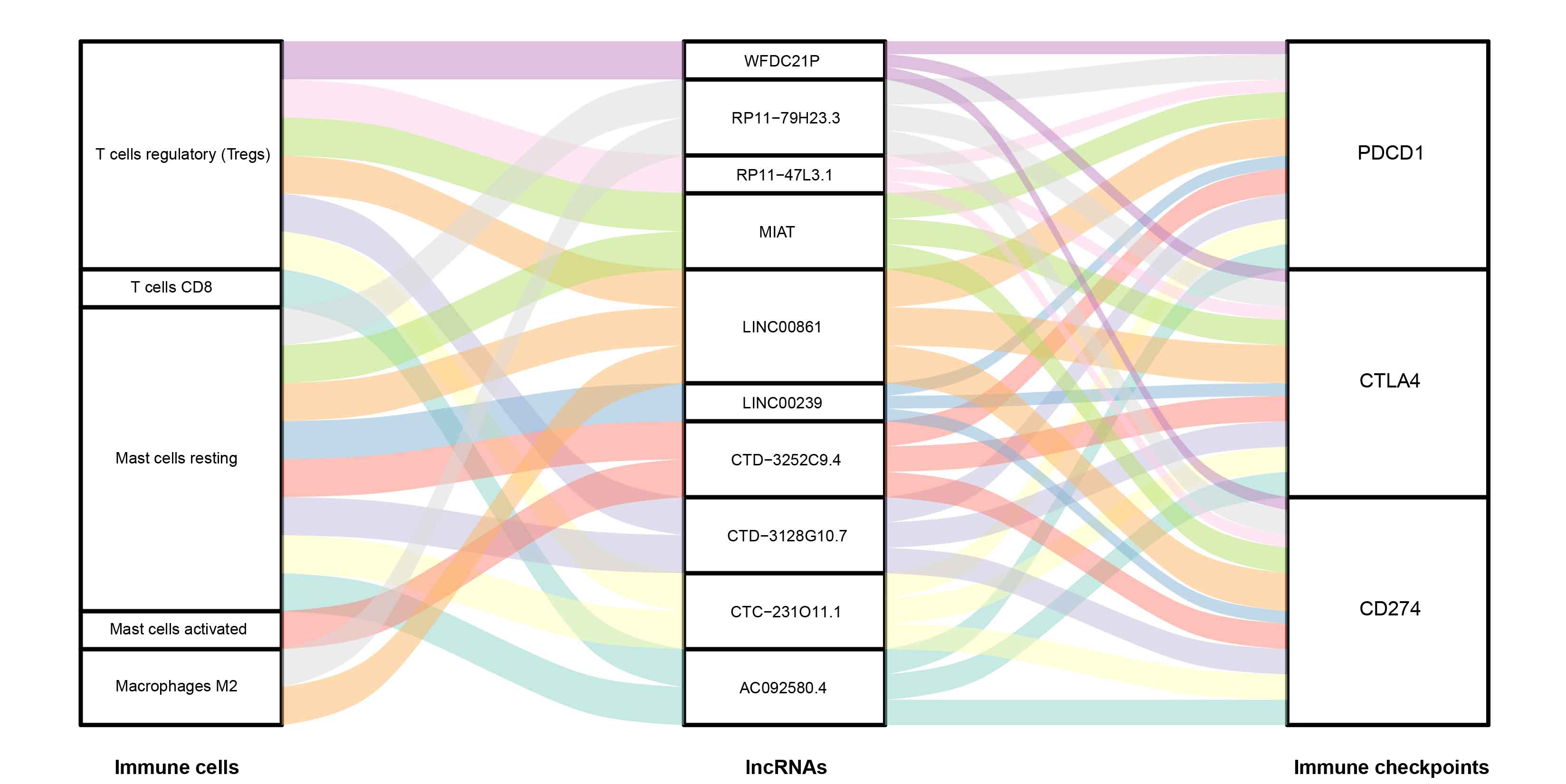
**

**Supplemental Figure 4.** The Sankey diagram shows the connections between lncRNAs, immune cells and actionable immune checkpoints.
